# Long-Chain Polyunsaturated Fatty Acids Effects on Cardiovascular Risk in Childhood: A Narrative Review

**DOI:** 10.3390/nu15071661

**Published:** 2023-03-29

**Authors:** Maria Elena Capra, Brigida Stanyevic, Antonella Giudice, Delia Monopoli, Nicola Mattia Decarolis, Susanna Esposito, Giacomo Biasucci

**Affiliations:** 1 Pediatrics and Neonatology Unit, Guglielmo da Saliceto Hospital, 29121 Piacenza, Italy; 2 Società Italiana di Nutrizione Pediatrica, 20126 Milan, Italy; 3 Pediatric Clinic, Department of Medicine and Surgery, University Hospital of Parma, 43126 Parma, Italy; 4 Department of Medicine and Surgery, University of Parma, 43126 Parma, Italy

**Keywords:** hypercholesterolemia, hypertriglyceridemia, long-chain polyunsaturated fatty acids, NAFLD, omega-3, pediatrics, overweight

## Abstract

Long-chain polyunsaturated fatty acids (LCPUFAs) are semi-essential fatty acids widely studied in adult subjects for their healthy-heart effects, especially on secondary prevention in patients who already experienced a cardiac event. LCPUFAs consumption is safe, without adverse effects, and they are usually well-tolerated; they can be taken either in foods or as nutritional supplements. LCPUFAs’ positive effect on global health has been worldwide recognized also for pediatric patients. In childhood and adolescence, research has mainly focused on LCPUFAs’ effects on neurodevelopment, brain and visual functions and on maternal–fetal medicine, yet their cardiovascular effects in childhood are still understudied. Atherosclerosis is a multifactorial process that starts even before birth and progresses throughout life; thus, cardiovascular prevention is advisable and effective from the very first years of life. Nutritional and lifestyle interventions are the main factors that can interfere with atherosclerosis in childhood, and the consumption of specific nutrients, such as LCPUFAs, can enhance positive nutritional effects. The aim of our narrative review is to analyze the effect of LCPUFAs on cardiovascular risk factors and on cardiovascular risk prevention in developmental age, focusing on specific conditions such as weight excess and dyslipidemia.

## 1. Introduction

Long-chain polyunsaturated fatty acids (LCPUFAs) can be classified as nutraceuticals, as they are nutrients that can have positive effects on human health. In the past decades, LCPUFAs have been widely studied in adult subjects for their healthy-heart effects, and promising results have been reached in cardiovascular prevention and treatment [1]. In addition, LCPUFAs have many positive effects in developmental age. In childhood and adolescence, research has mainly focused on LCPUFAs’ effects on neurodevelopment, brain and visual functions and on maternal–fetal medicine [2]. LCPUFAs healthy-heart actions in developmental age have been studied only in recent years, yet this seems to be an important issue, as LCPUFAs seem to exert positive cardiovascular actions in children and adolescents as well. The aim of our narrative review is to analyze LCPUFAs’ effects on cardiovascular risk prevention in children and adolescents.

## 2. Cardiovascular Risk in Developmental Age

**Figure 1:**
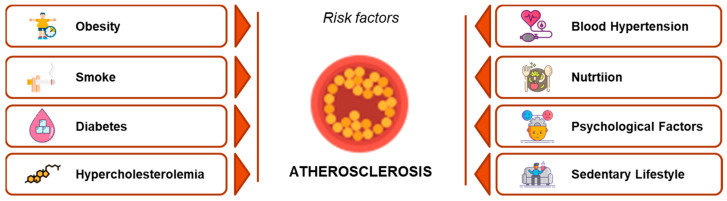
The main cardiovascular risk factors, derived from the INTERHEART Study data. Hypercholesterolemia is highlighted in red, as it is one of the most remarkable risk factors in pediatric age (adapted from reference [7]).

Coronary heart disease (CHD) is one of the major morbidity and mortality causes in Western countries [3]. CHD affects mainly adult subjects, but it is worldwide known and accepted that the atherosclerotic process starts before birth and progresses throughout childhood [4,5]. Atherosclerosis is a multifactorial process, and the exposure to conditions linked to increased cardiovascular risk accelerates and worsens the atherosclerotic cascade [6]. The main cardiovascular risk factors are summarized in Figure 1, which are adapted from the INTERHEART Study data [7].

CHD prevention is highly recommended and advisable starting from fetal age and throughout childhood and it can be performed at different stages, as shown in Table 1.

CHD risk factors can be present starting from birth, often on a genetic basis, or they can become evident in the following years of life both on a genetic and metabolic basis. CHD detection, treatment and risk stratification is fundamental in developmental age [8]. Hypercholesterolemia, especially in its familial form, is a cardiovascular risk factor already present, detectable and treatable from the first years of life, and an early and adequate treatment literally helps patients “gain decades of life” [9]. The main tools that can be used in CHD prevention in childhood are nutrition, lifestyle changes, nutraceuticals and pharmacological treatment, according to the extent and the type of risk factor that has to be treated. LCPUFAs are among the most widely studied nutraceuticals in cardiovascular prevention [8].

## 3. Long-Chain Polyunsaturated Fatty Acids (LCPUFAs)

Fatty acids are fat-soluble compounds consisting of hydrocarbon chains with a methyl group at one end and a carboxyl group at the other one. The biological activity of fatty acids is defined by the presence, number and position of each double bond present in each compound as well as by the length of the carbon chain itself [10]. Fatty acids are defined as “unsaturated” when they contain at least one double bond within their chain and “polyunsaturated fatty acids” (PUFAs) when two or more double bonds are present in the acyl chain. Otherwise, fatty acids are defined as “saturated” when they do not contain any double bonds [10]. PUFAs are metabolized in several tissues, although mostly in the liver [11].

### 3.1. Omega-3 and Omega-6 Series

**Figure 2:**
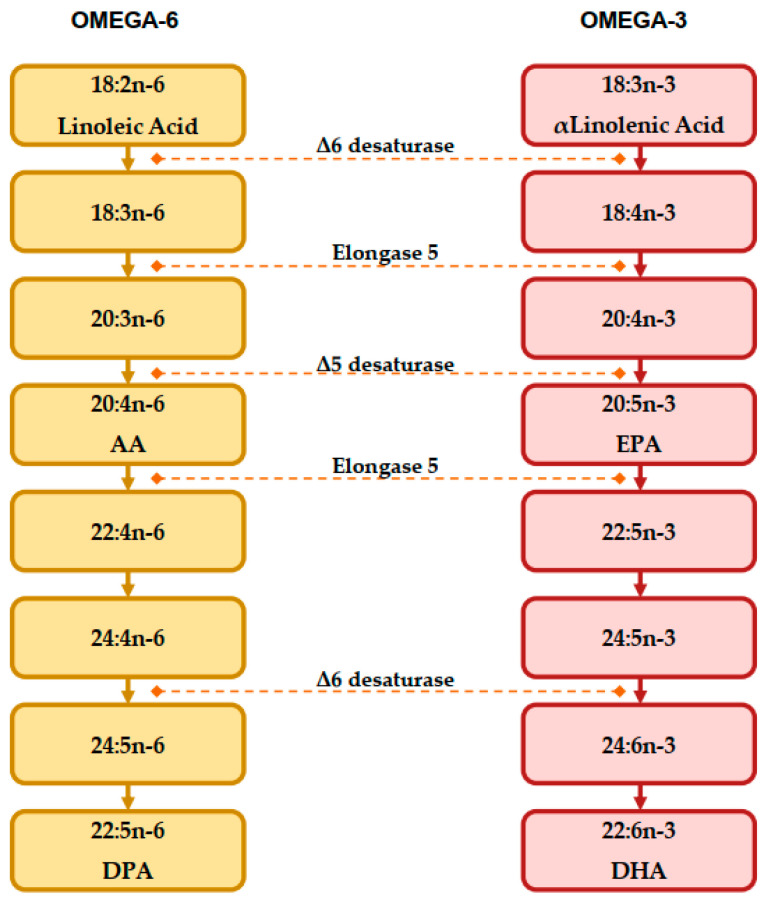
Biosynthesis pathway of LCPUFAs from precursors, adapted from Patterson et al. [12].

PUFAs can be subdivided in two families, both having important implications in human health: “omega-3” and “omega-6” series, according to the location of the last double bond to the methyl terminal of the molecule. Fatty acids that cannot be synthesized by humans are considered as “essential fatty acids”, and they must be introduced with diet. Essential fatty acids are linoleic acid (LA, C18:2, precursor of the *n*-6 series) and α-linolenic acid (ALA, C18:3, precursor of the *n*-3 series). As shown in Figure 2, LA and ALA are the “founder” members of each PUFA family, and as essential fatty acids, they need to be introduced with diet [12].

As mentioned above, LA is the precursor of omega-6 PUFAs, and ALA is the precursor of omega-3 PUFAs, both being progressively desaturated and elongated by the same enzymes [13,14,15,16]. Eicosapentaenoic acid (EPA), docosapentaenoic acid (DPA) and docosaexaehnoic acid (DHA) derive from ALA, and arachidonic acid (AA) derives from LA, respectively, and they are the most relevant fatty acids. These fatty acids are considered as “conditionally essential” because they can be synthesized by humans, but their synthesis depends on the relative availability of the respective substrate and on the efficacy of the converting enzymes [16,17]. In humans, this process seems to be insufficient; studies showed that in young men, the conversion rate of ALA to EPA and DHA was 8% and 4%, respectively, whereas in healthy young women, the conversion rate was 21% for EPA and 9% for DHA [18]. EPA can also be a substrate for the creation of DHA and vice versa, even if the conversion efficiency of EPA into DHA is <0.1% in adult males [19,20].

In the European population, the mean intake of fatty acids stands at 28–42% of the total daily energy consumed [21], whereas in the ancestral diet, the intake of these nutrients has been esteemed to be approximately 20–30% of the total energy [22,23]. Basically, during the last decades, industrialized societies have experienced a dramatic increase in the consumption of lipids, specifically saturated fats, omega-6 PUFAs and trans-fatty acids, as well as an overall decrease in omega-3 PUFAs intake [24]. As a result, in Western countries, the average LA dietary intake is 5 to 15 times higher than that of ALA [25]. Indeed, although the optimal omega-6 to omega-3 PUFAs ratio should be about 4:1, LA-derived fatty acids dietary intake is currently much more predominant in Western diets (omega-6/omega-3 ratio > 10:1). In addition, it has been observed that ALA intake significantly increases EPA and DPA levels, but there is a significantly lower increase for those of DHA in certain blood cell lines (white blood cells, red blood cells and platelets) and in breast milk [26].

As LA and ALA are not synthesized in animals, the first studies demonstrating the essentiality of these molecules were conducted on rats, and their deficiency caused a series of severe symptoms [27]. Several studies have shown that omega-3 and omega-6 PUFAs play important roles in membrane lipid composition: they can alter the blood lipid profile, influence gene expression, and interfere with eicosanoid biosynthesis and with cell signaling cascades [12,28,29]. Indeed, omega-6 and omega-3 PUFAs are fundamental for the synthesis of eicosanoids such as thromboxane (TX), prostaglandins (PGs), leukotrienes, prostacyclin (PGI), hydroxycosatetraenoic acid, hydroperoxytetraenoic acid and lipoxins, which play an essential role in vascular pathophysiology [30]. These eicosanoids are involved in different physiological actions, including vasodilation, vasoconstriction, pro/antiplatelet pro/anti-inflammatory effects, cell growth, cell proliferation and immune response. However, it is worth mentioning that the functions of EPA-derived PGs differ from those derived from AA. In fact, while AA-derived PGE2 and TXA2 promote inflammation and platelet aggregation as well as act as vasoconstrictors, PGE and TXA derived from EPA only act as vasodilators and anti-aggregators [31].

### 3.2. Nutritional Sources of LCPUFAs

In the human diet, the main ALA sources are vegetables, especially vegetable oils and some seeds and nuts [31]. Among vegetable oils, a good amount of ALA can be found in walnut, canola, soybean, linseed and echium seed oils. For example, linseed oil is very rich in ALA (49.2 g/100 g) [16]. A high amount of ALA can also be found in algae [13], paprika Capsicum annuum (30.27% in the Jariza variety and 29.93% in the Jaranda variety) [32], Trichosanthes kirilowii (33.77–38.66% of seed’s oils) [33], and chia Salvia hispanica (64.04% of seed’s oil FA and 16.4 g/100 g of ground chia seeds) [34,35].

As reported by the CREA, the Italian leading research organization dedicated to agri-food chains [36] (Table 2), high amounts of ALA are also found in other vegetable foods such as beans, dried lentils, nuts, maize, soya flour, wheat germ, garlic, oatmeal, pearl barley, buckwheat and various other ones. The ALA content in animal products seems to be generally lower, and the agri-food industry has further contributed to the depletion of omega-3 FAs in animal meat. Wild animals and birds that eat wild plants are very thin, and the fat content of their carcasses is 3.9% [37] with about five times more PUFAs content per gram than that found in domestic livestock [38]. Modern animal husbandry has emphasized the use of grains poor in omega-3 and rich in omega-6 to feed livestock, so domestic beef contains very small or insignificant quantities of ALA, whereas deer feeding on ferns and mosses contain more ALA in their meat [39].

Modern aquaculture also produces fish containing fewer omega-3 PUFAs than that naturally grown in rivers, lakes, sea and ocean [39]. Nevertheless, as shown in Table 3 and Table 4 [36], fish remains the primary source of EPA and DHA supply for humans [40]. This can be explained by the fact that many fish feed on algae rich in EPA and DHA [41,42]. Microalgae are considered as the main omega-3 LCPUFAs producers in the biosphere. For example, *Crypthecodinium cohnii* and *Schizochytrium* spp. contain 40% and 55% of the total FAs in the form of DHA, respectively, being the two main algal sources of DHA [43].

In addition, fish oils are rich in EPA and DHA. Cod liver oil contains 12.2% EPA, 12.7% DHA and 1.7% DPA [44], haddock oil contains 14.8% EPA, 24.8% DHA and 1.9% DPA, halibut oil contains 9.6% EPA, 30.6% DHA and 2.6% DPA [45], salmon oil contains 6.2% EPA, 9.1% DHA and 1.8% DPA [46]. In fish and fish oils, omega-3 LCPUFAs are mostly present as free FAs and triacylglycerides [47,48]. For these reasons, omega-3 supplements produced by the food industry are mainly derived from fish [49].

The appropriate daily intake values for the Italian population are 250 mg of EPA+DHA and 100 mg of DHA for subjects aged less than 18 years; LCPUFAs should account for 5–10% of total daily energy, of which omega-6 account for 4–8% and omega-3 account for 0.5–2%, respectively [50].

As previously mentioned, ALA is the precursor to EPA and DHA in the human body. However, this bioconversion is limited, and therefore, an adequate dietary intake of long-chain omega-3 is required. However, it should be underlined that it is strongly recommended to take omega-3 PUFAs also from other food sources as part of a balanced diet, since the frequent consumption of seafood can expose subjects to the neurotoxic effect of methyl mercury, which is highly detrimental for the development of the fetus’ central nervous system [51].

### 3.3. LCPUFAs in Pregnancy and Lactation

Human milk is known to contain LCPUFAs, mostly consisting of 0.5–0.6% AA and 0.2–0.3% DHA [52], providing approximately 7 mg DHA for every 100 mL over a 12-month lactation period [53]. DHA is well represented in the forebrain areas involved in processes and memory. Autopsy studies have revealed a higher forebrain DHA presence in breastfed infants compared to formula-fed non-supplemented infants [54,55]. Although a direct cause–effect relation could not be extrapolated, higher developmental scores observed in breastfed subjects have been linked to this DHA brain different concentration [56,57]. It has also been reported that maternal LCPUFAs intake during pregnancy increases the duration of pregnancy and reduces the frequency of preterm delivery [58] as well as the likelihood of the child suffering from asthma in adolescence [59].

The main results of studies on the effects of DHA supplementation in pregnant and lactating women have shown that breastfed infants appear to benefit from the presence of DHA in human milk if maternal supplementation is started during pregnancy. In fact, high-dose DHA supplementation initiated at mid-pregnancy in mothers has been associated with long-term positive effects on neurodevelopment and intelligence quotient scores [2]. Data from two large intervention trials showed the effects of high maternal intakes of DHA (ranging from 0.8 to 2.2 g/day) starting at 18–20 weeks of gestation and continued until childbirth [60] or up to 3 months postpartum [61]. These intakes were associated with higher child scores for hand–eye coordination at 2.5 years [60] and for cognitive functions at 4 [61] and 7 years [62]. A further study that tested DHA supplementation at the dose of 400 mg per day in pregnant women from the 18th week of gestation until delivery highlighted the problem of a baseline DHA deficiency in pregnant women. In fact, a higher risk of poor visual acuity has been found among infants born to women who did not take DHA supplements [63]. No improvement in visual performance was demonstrated with lower DHA dosages (200 mg/day) [64]. However, measurable and long-term functional effects on cognitive development have been associated with higher dosage DHA supplementations started at mid-pregnancy, whether or not they were prolonged during breastfeeding [65]. Given these data, milk companies have undertaken supplementation studies to explore the functional associations of dietary DHA supplementation in formula-fed infants. Here again, the infant’s DHA status at birth and genetic inheritance seem to play a major role [62].

## 4. Long-Chain Polyunsaturated Fatty Acids (LCPUFAs) in Cardiovascular Prevention in Adult Population

Omega-3 LCPUFA have been widely studied and used in cardiovascular risk prevention and treatment in the adult population. The administration of omega-3 LCPUFA has been proved to be safe and generally well tolerated [1]. EPA and DHA are used in adult subjects with hypertriglyceridemia to lower plasma triglyceride levels. A 2 to 4 g per day supplementation of EPA and DHA combination has been proven to be effective in reducing triglycerides-rich lipoproteins, in particular VLDL. EPA and DHA act through their interaction with peroxisome proliferator-activated receptors (PPARs), thus causing a reduction in Apolipoprotein B secretion, even if their overall mechanism of action is certainly much more complex and only partially understood [1]. Omega-3 effects on cardiovascular health have been studied since many decades. In the DART study published in 1989, a group of male subjects on secondary prevention after myocardial infarction showed a 29% reduction in mortality after a two-year treatment with omega-3 when compared to placebo [66]. In the Japan EPA Lipid Intervention (JELIS) study, EPA 1.8 g/day supplementation in adult patients on statin therapy (20% with history of coronary heart disease at baseline) led to a 19% risk reduction in major coronary events in a 4.6 years follow-up [67]. The GISSI study [68] can be considered a milestone in this field [68]: after a twelve-month treatment with omega-3, patients with recent myocardial infarction showed a 15% reduction in global mortality and cardiovascular mortality when compared to patients receiving placebo. The efficacy of high dose omega-3 supplementation on lowering serum triglycerides in adult patients has been confirmed in many recent studies [69,70,71], highlighting a reduction up to 45% of basal values, and in one metanalysis [1]. In adult subjects, many studies have been conducted on the effect of omega-3 on non-lipid cardiovascular disease parameters. In the Reduction of Cardiovascular Events with EPA-Intervention Trial (REDUCE-IT) [72], the authors evaluated the effect of EPA contained in fish oil on Atherosclerotic Cardiovascular Disease (ASCVD) outcomes in adult patients with hypertriglyceridemia. The 2 g/day EPA supplementation resulted in a 25% reduction in relative risk of major cardiovascular events when compared to placebo. These findings were confirmed by the EVAPORATE trial, in which patients supplemented with high-dose of icosapent ethyl (with respect to those receiving a placebo with the same mineral oil comparator used in the REDUCE-IT) had a reduction in atherosclerotic plaque progression [73]. In the STRENGHT study, a study to assess long-term outcomes of Statin Residual Risk with Epanova in High Cardiovascular Risk Patients with Hypertriglyceridaemia, the 4 g daily consumption of EPA and DHA was not effective in reducing triglycerides levels [74]. In a recently published EAS document [75], the discrepant results of these two studies were attributed to the different choice of comparator (the mineral oil comparator used in the REDUCE-IT study could cause a collateral increase in LDL-C and Apolipoprotein B, whereas the corn oil used in the STRENGHT study is neutral), to the different formulations used and to the differences in the patient populations enrolled (higher percentage of patients with established coronary artery disease in the REDUCE-IT). Therefore, in the EAS document, it is recommended to be cautious when prescribing omega-3 supplements for cardiovascular risk reduction, paying attention also to the fact that atrial fibrillation’s incidence was higher in the intervention groups in both studies [75]. In the VITamin D and OmegA-3 TriaL (VITAL) study, a large-scale randomized trial, omega-3 supplementation’s effect on cardiovascular disease prevention was tested in the general population, which was unselected for increased CHD risk. In this study, supplementation with 1 g/day of omega-3 (1.2:1 ratio of EPA to DHA) and 2000 UI/day of vitamin D3 for 5.3 years significantly reduced total myocardial infarction, fatal myocardial infarction and recurrent hospitalization for heart failure when compared to the group receiving placebo (olive oil) [76]. In a recent document of the European Society of Cardiology (ESC), it has been suggested that additional studies are needed to better clarify which category of subjects may be more likely to benefit from omega-3 supplementation [77]. Studies analyzing the effect of omega-3 on total mortality and on cardiovascular risk showed no significant effect but only a suggestion that omega-3 LCPUFAs may reduce CHD [78]. In particular, the omega-3 supplementation of pediatric patients with attention deficit hyperactivity disorder resulted in a positive effect on functional outcome even if the available evidence is not sufficient to recommend omega-3 supplementation. A transient benefit was also highlighted in patients with cystic fibrosis. Further studies are already ongoing and will be certainly needed in this field also because the dose of omega-3 to be administered is a very relevant issue to obtain the desired outcome.

## 5. Long-Chain Polyunsaturated Fatty Acids (LCPUFAs) in Cardiovascular Prevention in the Pediatric Population

LCPUFAs’ effects in developmental age have been widely studied. In a recent multicenter, randomized double-blind controlled study aimed at investigating the effect of DHA supplementation on metabolic markers of obese children, children receiving DHA showed a remarkable increase in DHA plasma levels, which could have an anti-inflammatory effect [79]. In another study, it has been postulated that the presence of high-plasma omega-3 LCPUFA values may exert positive effects in terms of visual–spatial attention mechanism in reading and writing functions [80]. In a multicentric trial, supplementation with up to 7 mg/kg/day of DHA did not improve neurological functions in children with phenylketonuria [81].

LCUPFAs’ effect on cardiovascular prevention in developmental age has been recently studied, even if it has been less investigated than in adult subjects. ESPGHAN has recently stated the positive effect of LCPUFAs on global health in the pediatric population [82]. We will analyze the main conditions and cardiovascular risk factors that seem to be affected by omega-3 supplementation.

### 5.1. Non-Alcoholic Fatty Liver Disease

In children aged below 18 years, Non-Alcoholic Fatty Liver Disease (NAFLD) is defined as a chronic hepatic fat accumulation that is not due to genetic disorders, drugs affecting liver function, infections or ethanol consumption [83]. It represents the most common cause of chronic liver disorders in developmental age, especially in Western countries [84]. NAFLD is strongly associated with obesity, insulin resistance, hypertension and dyslipidemia [85]. For this reason, it can be considered the hepatic manifestation of the metabolic syndrome [86], which consists, based on the International Diabetes Federation (IDF) criteria, of a combination of abdominal obesity with two or more other clinical features, including high blood pressure, elevated triglycerides, low HDL cholesterol, and hyperglycemia [87]. Therefore, NAFLD can be considered as a remarkable cardiovascular risk factor in particular regarding early atherosclerotic changes, systolic and diastolic dysfunction, high blood pressure and cardiac hypertrophy [88]. For adult subjects, the definition of NAFLD has been recently updated to that of metabolic dysfunction-associated fatty liver disease (MAFLD) [89]. An international panel of experts has recently proposed diagnostic criteria for MALFD in pediatric subjects even if it is still debated to include the Homeostasis Model Assessment Insulin Resistance (HOMA-IR) and C Reactive Protein values among the diagnostic criteria [90]. In overweight children, the prevalence of NAFLD can be estimated at around 31.6% [91], whereas MAFLD in European overweight children and adolescents may account for 24.2% [90,92]. NAFLD includes two different histological patterns: NAFL (Non-Alcoholic Fatty Liver), which is identified by a simple steatosis in 5% or more hepatocytes, and NASH (Non-Alcoholic Steato-hepatitis), which is associated with lobular inflammation and hepatocellular damage with or without fibrosis [93]. If not promptly detected and treated, the natural history of NAFLD consists of a progression from NAFL to NASH, which in turn can evolve into cirrhosis and hepatocellular carcinoma [94]. Multiple risk factors are involved in the development and progression of NAFLD. Certainly, obesity and sedentary lifestyle are the most significant ones, which are followed by genetic, metabolic, epigenetic and environmental factors, and gut microbial dysbiosis [95]. According to the “multiple-hit-hypothesis”, pediatric NAFLD pathogenesis is a complex process, where the main actors are represented by fat accumulation, lipotoxicity, liver inflammation and oxidative stress [96,97,98]. NASPGHAN and ESPGHAN recommend screening for NAFLD in obese children between 9 and 11 years of age, and in overweight children with additional risk factors such as insulin resistance, dyslipidemia or a family history of NAFLD. As screening tool, NASPGHAN guidelines recommend the assessment of serum alanine aminotransferase (ALT), whereas ESPGHAN recommends an ultrasound scan in association with ALT [99]. However, the gold standard for the diagnosis of NAFLD still remains the liver biopsy for histological evaluation, despite its invasive nature [100]. Currently, an approved pharmacological therapy for pediatric NAFLD is still missing, and the main treatment is represented by an improvement of diet and physical activity [101]. Several studies, also in consideration of the etiopathogenesis of this liver disorder, have been performed with the aim to find pharmacotherapy strategies and dietary supplementations, such as antioxidants (vitamin E), insulin sensitizers (metformin), ursodeoxycholic acid (UDCA) and probiotics. Omega-3, including DHA and EPA, have been studied as NAFLD treatment in developmental age [102,103]. Indeed, LCPUFAs may play a pivotal role in pediatric metabolic syndrome through epigenetic effects (including miRNA, histone acetylation and DNA methylation), thus influencing the expression of genes involved in inflammatory and other metabolic pathways, which are essential for the metabolic syndrome induction [104]. In this section, we focus on the role of LCPUFAs in children with diagnosis of NAFLD, with the purpose of investigating their possible beneficial effects. LCPUFAs, owing to their biological mechanisms, have a role in reducing inflammation pathways and in regulating nuclear transcription factors involved in liver lipid metabolism and adipose tissue function, which are altered in NAFLD [105]. Nobili et al. performed a double-blind randomized controlled trial on 60 children, with biopsy-proven NAFLD, referred to the Liver Unit of the Bambino Gesù Pediatric Hospital in Rome (Italy). They showed that a 6-month DHA supplementation improves liver fat content detected by ultrasonography, increases insulin sensitive index and decreases plasma triglycerides levels, regardless of the DHA supplementation dose (250 mg/day or 500 mg/day). However, they did not report long-lasting effects on ALT and body mass index (BMI) following DHA supplementation [106]. Jules et al. performed a cross-sectional analysis, as a part of the Treatment of Non-Alcoholic Fatty Liver Disease in Children (TONIC) trial and the NAFLD database study, to evaluate fish intake and omega-3 fatty acids intake and their effect on ALT serum levels and liver histological features in pediatric patients with NAFLD. Their results showed that children with NAFLD consume a lower amount of omega-3 fatty acids than recommended and that a higher fish and omega-3 fatty acids intake is associated with a reduction in ALT values and with a reduction in both portal and lobular liver inflammation detected after liver biopsy [107]. Even if specific recommendations about fish and omega-3 fatty acids intake in the young population with NAFLD are still unavailable, the majority of authors suggest consuming at least two portions (approximately 224 g) of fish per week [108]. Boyraz et al. carried out a randomized trial analyzing the effect of omega-3 treatment (1000 mg of PUFAs once daily) for 12 months in 56 obese Turkish children with NAFLD. They found that children who received PUFAs had an improvement in insulin sensitivity, systolic blood pressure, fasting glucose, ALT and aspartate aminotransferase (AST) levels, and triglycerides values compared to the placebo group. Furthermore, they reported an ultrasonography amelioration of fat liver content in the intervention group [109]. Another randomized controlled study, carried out by the expert group from the Hepato-Metabolic Department of the Bambino Gesù Pediatric Hospital in Rome, established beneficial outcomes of the dietary supplementation with DHA (500 mg) and vitamin D (800 UI) orally once a day for 24 weeks in children with NAFLD histological diagnosis. Triglycerides, ALT and insulin resistance decreased with the mixture treatment and reduction in hepatic stellate cells (HSC) activation, and fibrillar collagen was evident at histological examination [110]. LCPUFAs, through liver and abdominal visceral fat improvement, can also reduce cardiovascular risk in overweight children with NAFLD, as observed in a randomized trial in which NAFLD young patients were treated with DHA (250 mg/day) for a six-month period. Moreover, in the DHA group, a positive trend for fasting insulin and triglycerides serum levels was described [111]. In contrast, in a study carried out in four Pediatric Departments in Poland on patients with NAFLD aged 11–15 years, the use of omega-3 in a young population with NAFLD was not recommended. The authors found that omega-3 fatty acid supplementation (DHA and EPA 450–1300 mg/day) for 6 months did not decrease ALT levels and did not lead to any improvement in ultrasound detected liver steatosis, even if AST and gamma-glutamyl transpeptidase (GGT) serum concentrations were reduced [112]. In conclusion, the majority of available trials suggest that omega-3 fatty acids supplementation should be considered as a strategy for pediatric NAFLD due to their significant positive effects on hepatic fat content, insulin resistance, lipid levels and histological pathways (Table 5). LCPUFAs supplementation should not be used as a single strategy but as a complement of nutritional and lifestyle interventions, which represent milestones in the cardiovascular disease prevention in developmental age [113]. Further studies are needed to evaluate long-term outcomes of LCPUFAs supplementation in pediatric patients with NAFLD and consequently on the associated cardiovascular diseases.

### 5.2. Hypercholesterolemia

Pederiva et al. reported on the short-term use of nutraceuticals, in association with nutritional treatment, for the control of the cardiovascular risk progression, starting from infancy. Supplementation with omega-3 LCPUFAs, in particular DHA, is able to improve plasma HDL cholesterol levels in pediatric patients with FH [114]. Unfortunately, the use of nutraceuticals in pediatric patients with hypercholesterolemia is still debated because there are scarce and contrasting results about their long-term efficacy and safety in pediatric age. Their use is of particular interest in pediatric patients with familial hypercholesterolemia (FH), one of the most common inherited diseases, involving approximately 1 out of 250 individuals in the general population [9]. In a double-blind, placebo-controlled, randomized study (EARLY study), the effect of a six-week DHA supplementation (1.2 g/day) was analyzed in a cohort of pediatric patients with familial hypercholesterolemia (FH). In the intervention group, endothelial-derived flow-mediated dilation of the brachial artery (a surrogate atherosclerosis marker) increased significantly with respect to the control group, demonstrating that DHA supplementation may have a positive effect on endothelial function, thus preventing the progression of early CHD in high-risk children, such as those with FH [115]. Barkas et al. showed that omega-3 fatty acids supplementation might lead to a reduction in TC in patients with FH. In this study [116], the authors demonstrated no impact on HDL-C levels in FH individuals and a non-significant trend (likely due to the small sample size and design of the study) in TC and LDL-C reduction, supporting the conflicting evidence regarding the impact of omega-3 fatty acids on cholesterol [117,118]. In another trial, a significant non-HDL-C and apoB reduction was highlighted in subjects who followed a diet rich of icosapent ethyl, which is a highly purified EPA [119].

In a systematic review and meta-analysis, fish oil supplementation considerably reduced BMI but not TC, HDL-C and LDL-C serum levels in obese children [120]. In a pilot study carried out in children and adolescents, an emulsified combination of plant sterols, fish oil and group-B vitamins resulted in lower levels of the atherogenic lipoprotein VLDL, IDL-1 and IDL-2 subfractions [121]. The putative mechanism of this cholesterol-lowering effect may be the competition between intestinal plant sterols/stanols and intestinal cholesterol absorption in mixed micelles and an overexpression of the enterocyte transport proteins [122]. This complex interaction has been seldom explored in children and adolescents [123]; thus, further studies are needed to investigate the effect of omega-3 LCPUFAs on the lipid profile in pediatric patients with hypercholesterolemia in order to perform a preventive and individualized therapeutic intervention.

### 5.3. Hypertriglyceridemia

Hypertriglyceridemia, especially in its mild and moderate form, is a very common dyslipidemia in childhood and adolescence, involving up to 10% of children in the general population [124,125,126]. Primitive forms of hypertriglyceridemia are genetically determined usually rare conditions, and patients suffering from these conditions need to be referred to a Pediatric Lipid Clinic for tailored and strict nutritional counseling and clinical follow-up [114]. The most common cause of mild or moderate hypertriglyceridemia in childhood is secondary to weight excess, which leads to the development of insulin resistance and to altered lipid metabolism [127]. The National Expert Panel on Cholesterol Levels in Children and the Expert Panel on Cardiovascular Health Risk Reduction in Children stated normal lipid values in childhood [6]. However, triglycerides levels are better stratified in the 2010 guidelines of the Endocrine Society [128], as shown in Table 6.

Hypertriglyceridemia is one of the criteria of metabolic syndrome, and it is also correlated to the increase in cardiovascular risk and acute pancreatitis [129,130]. The first-line treatments for patients with hypertriglyceridemia are nutritional intervention and lifestyle change. It is important to increase daily physical activity, reduce caloric intake and replace simple sugars with complex carbohydrates. Furthermore, in the presence of severe hypertriglyceridemia, it is recommended to follow a very low-fat diet (less than 10% of fat) [131]. Pharmacotherapy represents the second-line intervention. The use of fibrates and niacin is widely studied among adults but not among children. The few studies available are not recent and not very reassuring. For instance, a multicenter study on the use of niacin in pediatric patients with hypercholesterolemia did not show any triglycerides and HDL levels reduction. In addition, some children had to drop out of the study because of significant adverse reactions [132]. Considering that there are few studies of pharmacotherapy in pediatric age, the use of omega-3 LCPUFAs seems to be a promising strategy [133]. Most of the studies considered fish oil supplements rich in omega-3 LCPUFAs even though alternative sources, such as nut oils or vegetable oils, can be considered as well [134]. Omega-3 LCPUFAs exert their cardio-protective role through a reduction in plasma triglycerides levels, thus achieving an anti-inflammatory effect through the regulation of transcription factors, membrane fluidity and gene expression [135] In a small study conducted in Slovakia involving 25 participants (mean age 16 years), patients were given an emulsified preparation containing plant sterol esters (1300 mg), fish oil (1000 mg eicosapentaenoic acid and 1000 mg docosahexaenoic acid), vitamins B12 and B6, folic acid and coenzyme Q10 daily for 16 weeks. After 16 weeks, a significant reduction in triglycerides levels was highlighted in the group of children between 10 and 16 years (baseline mean triglycerides level 1.1 mmol/L) [121].

Barkas et al. conducted a metanalysis on the effects of plasma lipid reduction on cardiovascular risk. The results of 17 trials, performed in adults and children, have been collected. Supplementation with of omega-3 LPUFAs led to a reduction in triglycerides and total cholesterol plasma levels in patients with hypercholesterolemia [116]. Omega-3 LCPUFAs supplementation had a positive effect on the triglycerides plasma level in pediatric patients with insulin resistance. In a randomized controlled trial involving 201 obese children with insulin resistance, the effects on lipid profile of omega-3 LCPUFAs and of metformin were compared. Triglycerides levels were significatively lower in the group treated with omega-3 LCPUFAs [136]. However, there are studies in the literature that have demonstrated a clinically relevant but not statistically significant reduction in triglycerides levels and triglycerides/HDL ratio associated with the dietary supplementation of LCPUFAs in pediatric patients. In a retrospective study conducted in pediatric patients with dyslipidemia in Toronto, fish oil (at a dosage of 500 mg <10 years and 1000 mg >10 years) supplementation was compared to placebo: in the intervention group, no significative variation in lipid profile was found [137]. In another study conducted by De Ferranti et al., the effect on lipid profile of an omega-3 supplement was evaluated in healthy children and adolescents, aged 10 to 19 years, with moderate to severe hypertriglyceridemia recruited from Boston Children Hospital and community pediatricians. Omega-3 LCPUFAs were administered for a six-month period: though well tolerated by pediatric patients, they did not lead to a significant reduction in triglyceride levels at 3 and 6 months follow up [138]. In conclusion, LCPUFAs’ effect on pediatric patients with hypertriglyceridemia is still debated, and further studies are needed to verify the effect of these nutraceuticals on triglycerides levels.

### 5.4. Blood Pressure

Most studies have focused mainly on the influence that LCPUFAs have on visual and cognitive development, but only few have investigated the possible relationship with blood pressure in pediatric population prior to the study by Forsyth et al. in 2003. In their follow-up study, 147 infants born at term were subdivided in two groups, 71 infants in the LCPUFAs supplementation group and 76 infants in the non-supplementation group, and each child was fed with the corresponding test formula during the first four months of life. Data collected at 6 years of age revealed that the LCPUFAs group showed significantly lower mean blood pressure (95% confidence interval—0.5 to 5.4 mmHg; mean difference—3.0 mmHg) and diastolic blood pressure (confidence interval—0.6 to 6.5 mmHg; mean difference—3.6 mmHg) compared to the group that did not receive supplementation. In addition, the LCPUFAs group showed blood pressure values comparable to the reference group of 83 breastfed infants. Despite the various limitations of the study, the authors concluded that dietary intake of LCPUFAs during the first months of life appears to be associated with lower blood pressure in later childhood. Consequently, since blood pressure trends originate from childhood, early supplementation of LCPUFAs in the diet may decrease cardiovascular risk in adulthood [139].

## 6. Conclusions

LCPUFAs exert positive effects on cardiovascular risk factors in developmental age, especially in subjects with dyslipidemia and with NAFLD. Their intake is safe and presents no adverse effects, and their positive effect on global health has been worldwide recognized in pediatric patients. A limitation to the evaluation of LCPUFAs’ healthy-heart effects in developmental age is that LCPUFAs doses and the length of their administration are not standardized and may vary greatly form one study to another. As demonstrated in studies conducted in adult subjects, a high dosage of LCPUFAs is often necessary to obtain a positive modification of cardiovascular risk factors. However, studies in adult subjects often focus on secondary prevention, whereas pediatric studies mainly focus on primordial or on primary prevention; therefore, lower dosages given for longer periods of times may have positive effects as well. In conclusion, LCPUFA’s effect on cardiovascular risk factors in developmental age seems to be promising, but further studies are needed to better define the specific effects of different LCPUFAs intakes on various CHD risk factors.

## Figures and Tables

**Table 1:** Types of prevention.

Different Types of Prevention	
Primordial prevention	Aimed at preventing risk factors
Primary prevention	Aimed at early identification and treatment of risk factors
Secondary prevention	Aimed at reducing the risk of other CHD events in subjects who have already had CHD events

**Table 2:** α-Linolenic acid (ALA) content of some plant and animal foods.

Food	G of Lipids per 100 g of Food	ALA % of Total Lipids
Beans, dried, raw	2	33.33
Lentils, dried, raw	1	12.82
Walnuts, dried	68.1	11.89
Soya oil	99.9	8.01
Wheat germ Oil	99.9	5.72
Sole, fresh	1.4	5.68
Trout, fresh	3	3.81
Lamb, lean only, raw	8.8	2.62
Milk, cow, partially skimmed	1.5	2.11
Beef, rib, lean only	6.1	2.10
Pecan Nuts	71.8	1.88

**Table 3:** Eicosapentaenoic acid (EPA) content of some fish/seafood and animal meat.

Food	G of Lipids per 100 g of Food	EPA % of Total Lipids
Squid, fresh	1.7	18.24
Sole, fresh	1.4	17.86
Octopus, fresh	1	17.82
Turkey, whole, with skin, raw	6.9	13.61
Sea bass, fresh	1.5	8.57
Salmon, fresh	12	8.43
Cod, deep frozen, raw	0.6	6.94
Trout, fresh	3	5.71
Beef, hind part cuts	3.4	2.74
Beef, in jelly, canned	1.8	2.71
Horse, lean only	1	1.27
Goat, lean only	2.3	1.09
Swine, light, leg	3.2	1.03

**Table 4:** Docosahexaenoic acid (DHA) content of some fish/seafood and animal meat.

Food	G of Lipids per 100 g of Food	DHA % of Total Lipids
Cod, deep frozen, roasted in oven	0.9	38.65
Tuna, fresh	8.1	26.54
Sole, fresh	1.4	25.97
Trout, deep frozen	2.3	22.98
Octopus, fresh	1	21.78
Salmon, fresh	12	11.27
Liver, chicken, raw	6.3	4.78
Beef, front part cuts	7	1.50
Chicken egg, whole, powder	36.4	0.81

**Table 5:** Studies on the LCPUFAs’ effects in pediatric Non-Alcoholic Fatty Liver Disease.

Type of Study	Population	Intervention	Results	Author
Randomized controlled trial	60 children with NAFLD diagnosis Age 8–12 years	DHA 250 mg/day or 500 mg/day for 6 months	- Reduction in liver fat content (US) - Improvement of insulin sensitivity	Nobili et al., 2011 [106]
Cross-sectional analysis	223 children with NAFLD diagnosis 8–17 years	To evaluate fish and omega-3 fatty-acids intake and their effects on ALT and liver histological characteristics	- Lower than recommended LCPUFAs intake - Higher LCPUFAs intake improves portal and lobular inflammation	St-Jules et al., 2013 [107]
Randomized trial	108 obese children with NAFLD diagnosis 9–17 years	LCPUFAs 1000 mg/day for 12 months	- Reduction in fasting glucose, triglycerides, AST, ALT, insulin resistance - Improvement of liver ultrasound features	Boyraz et al., 2015 [109]
Randomized trial	60 children with NAFLD 4–16 years	DHA (500 mg/day) and Vitamin D (800 UI/day) for 24 weeks	- Improvement of insulin resistance, triglycerides, ALT - Reduction in HSC activation and fibrillar collagen	Della Corte et al., 2016 [110]
Randomized trial	51 children with NAFLD diagnosis <18 years	DHA 250 mg/day for 6 months	- Improvement of liver and abdominal visceral fat (MRI) - Improvement of insulin and triglycerides levels	Pacifico et al., 2015 [111]
Randomized controlled trial	76 overweight/obese children with NAFLD diagnosis Median age 13	DHA and EPA 450–1300 mg/day for 6 months	- No ALT reduction - No liver ultrasound amelioration - Improvement of AST and GGT levels	Janczyk et al., 2015 [112]

DHA: docosahexaenoic acid, EPA: eicosapentaenoic acid, LCPUFAs: long-chain polyunsaturated fatty acids, US: ultrasound, MRI: Magnetic Resonance Imaging, ALT: alanine transaminase, AST: aspartate aminotransferase, GGT: gamma-glutamyl transpeptidase, HSC: hepatic stellate cells.

**Table 6:** Triglycerides levels stratification in pediatric age.

Age	Normal	Borderline	High	Very High	Severe	Very Severe
0–9 years	<75	≥75–99	≥100–499	≥500–999	≥1000–1999	≥2000
10–19 years	<90	≥90–129	≥130–499	≥500–999	≥1000–1999	≥2000

## Data Availability

Not applicable.

## Abbreviations

AA	Arachidonic Acid
ALA	Alfa-Linolenic Acid
ALT	Alanine AminoTransferase
Apo-B	Apolipoprotein B
ASCVD	Atherosclerotic Cardiovascular Disease
AST	Aspartate AminoTransferase
BMI	Body Mass Index
CHD	Coronary Heart Disease
DHA	DocosaHexaenoic Acid
DNA	DesoxyriboNucleic Acid
DPA	DocosaPentaenoic Acid
EPA	EicosaPentaenoic Acid
ESPGHAN	European Society for Paediatric Gastroenterology Hepatology and Nutrition
FA	Fatty Acid
FH	Familial Hypercholesterolemia
GGT	Gamma-Glutamyl Transpeptidase
HOMA.IR	Homestasis Model Assessment Insulin Resistance
HDL	High-Density Lipoprotein
HDL-C	High-Density Lipoprotein-Cholesterol
HSC	Hepatic Stellate Cells
IDF	International Diabetes Federation
IDL-1	Intermediate Density Lipoprotein 1
IDL-2	Intermediate Density Lipoprotein 2
LA	Linoleic Acid
LCPUFAs	Long-Chain Polyunsaturated Fatty Acids
LDL	Low-Density Lipoproteins
LDL-C	Low-Density Lipoproteins-Cholesterol
MAFLD	Metabolic dysfunction-Associated Fatty Liver Disease
miRNA	micro RiboNucleic Acid
MRI	Magnetic Resonance Imaging
NAFL	Non-Alcoholic Fatty Liver
NAFLD	Non-Alcoholic Fatty Liver Disease
NASH	Non-Alcoholic SteatoHepatitis
NASPGHAN	North American Society for Pediatric Gastroenterology, Hepatology and Nutrition
PGs	ProstaGlandins
PGI	Prostacyclin
PPAR	Peroxisome Proliferator-Activated Receptor
PUFAs	PolyUnsaturated Fatty Acids
TAG	Triacylglyceride
TC	Total Cholesterol
TONIC	Treatment Of Nonalcoholic fatty liver disease In Children
TX	ThromboXane
UDCA	UrsoDeoxyCholic Acid
VLDL	Very Low Density Lipoprotein
